# Investigations into the Geometric Calibration and Systematic Effects of a Micro-CT System

**DOI:** 10.3390/s24165139

**Published:** 2024-08-08

**Authors:** Matthias Hardner, Frank Liebold, Franz Wagner, Hans-Gerd Maas

**Affiliations:** Institute of Photogrammetry and Remote Sensing, TUD Dresden University of Technology, 01069 Dresden, Germany

**Keywords:** X-ray computed tomography, system calibration, measurement uncertainty, instrument geometry

## Abstract

Micro-Computed Tomography (µCT) systems are used for examining the internal structures of various objects, such as material samples, manufactured parts, and natural objects. Resolving fine details or performing accurate geometric measurements in the voxel data critically depends on the precise calibration of the µCT systems geometry. This paper presents a calibration method for µCT systems using projections of a calibration phantom, where the coordinates of the phantom are initially unknown. The approach involves detecting and tracking steel ball bearings and adjusting the unknown system geometry parameters using non-linear least squares optimization. Multiple geometric models are tested to verify their suitability for a self-calibration approach. The implementation is tested using a calibration phantom captured at different magnifications. The results demonstrate the system’s capability to determine the geometry model parameters with a remaining error on the detector between 0.27 px and 0.18 px. Systematic errors that remain after calibration, as well as changing parameters due to system instabilities, are investigated. The source code of this work is published to enable further research.

## 1. Introduction

**Figure 1:**
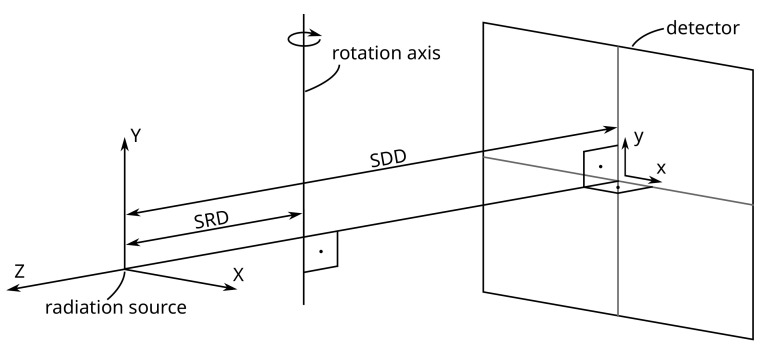
Ideal geometry of a computed tomography system without any misalignment.

The reconstruction of the 3D geometry using projections captured with X-ray computed tomography instruments depends on a known system geometry that describes the relation between all elements of the system. The elements of a cone beam µCT system consist of the X-ray source, the rotation axis for the sample, and the detector. The Source Detector Distance (SDD) lies on the optical axis, ideally intersecting the detector in the center, and is perpendicular to it. The rotation axis is parallel to the *Y*-direction of the detector plane and intersects with the optical axis at a 90° angle (Figure 1). The distance between the radiation source and the rotation axis is called the Source Rotation Axis Distance (SRD). The object is rotated on the rotation axis in constant steps, and at each rotation angle (or gantry angle), a projection is captured. Deviations from this ideal configuration are common and can significantly degrade the quality of the reconstructed images, resulting in blurred geometries and the loss of fine details.

This section provides a brief literature overview, highlighting the impact of misalignment on the reconstruction quality and examining the different approaches to the calibration of µCT systems from recent years. Additionally, we summarize the contributions of this study to the field.

### 1.1. Effect of Misalignment

Misalignment can be present in the different components of a system, and the effects on the reconstruction have been investigated in numerous publications. Ferrucci et al. [1] looked at the influence on detector angular misalignment based on simulated data. The errors in the 3D volume and on the detector frame were evaluated, and an improvement of the error using corrected projections was shown. Ametova et al. [2] presented a method for estimating the impact of the geometric misalignment on the measurement and tested it on simulated and experimental data. The estimation was based on the forward and back projections of surface points from multiple steel spheres. A more computationally demanding method was used in [3], where a Filtered Back Projection (FBP) was computed based on a Monte Carlo simulation with varying geometric parameters. This allowed insights into the effect of uncertainty of the geometric parameters to the reconstruction. The traceability of uncertainties for CT systems considering multiple influence factors was extensively reviewed by Dewulf et al. [4].

The precision requirements for geometric µCT calibration vary based on the object and the specific application of the reconstruction. Enhanced precision is critical when employing sub-voxel accuracy measurement techniques such as 3D-LSM [5], which enable deformation analysis in multi-temporal voxel data with precision ranging from 0.01 to 0.1 voxels. These techniques have been applied, for instance, to detect cracks in concrete samples during civil engineering material tests [6]. High precision is also essential to depict fine details at high magnification, such as when capturing individual carbon fiber strands or plant cells.

### 1.2. Offline Calibration

The misalignment can be modeled using a number of parameters, which can be determined by means of a calibration. Note that calibration in this context means the determination of parameters describing the geometry of the CT system. A large number of publications have been written on cone beam CT calibration in the last years. Extensive literature reviews can be found in [7,8]. For the definition of the system geometry, many different approaches and notations were pursued. Ferrucci et al. [7] used nine parameters to describe the geometry. Here, the misalignment of the detector and the rotation axis was modeled. In more recent publications, seven instrument parameters for the geometry were used, which fully describe the misalignment by the translation and rotation of the detector and the distance between radiation source and rotation axis SRD [2,9]. In [10], the pixel size was also included in the list of parameters.

The calibration can be conducted independent of the actual measurement by capturing multiple projections of a calibration phantom, which is also called offline calibration. In this case, the determined parameters must be applicable to the subsequent measurement, requiring a stable system geometry between the measurement and calibration. These calibration phantoms often consist of a number of steel spheres [2,9,11] and sometimes ball bearings [12,13] that are arranged on a cylindrical object. In [14], the steel spheres were arranged on an aluminum plate. Neuschaefer-Rube et al. [15] used a thin invar foil with a grid of holes, for which the coordinates were determined using an optical coordinate measuring machine. As in [9], tactile coordinate measuring machines were also used to determine the 3D coordinates before the calibration.

If the coordinates are optimized within the calibration process, the terms auto-calibration [8,10] or self-calibration [12] are used. Obviously, a large advantage of these self-calibration approaches, which are also well-established in photogrammetry [16], is that they avoid the difficult provision of highly accurate reference coordinate for the calibration object. Generally, initial estimates are required for all unknown parameters, including the 3D coordinates. A method requiring no initial estimates was discussed in [8] to determine the projection geometry of a µCT system.

To achieve different magnifications using computed tomography, the rotation axis can be adjusted in the Z-direction, changing the SRD. For the measurement of objects with varying sizes, a calibration would be necessary for each position of the rotation axis. Bircher et al. [17] built a µCT system with multiple interferometers and other sensors to determine the geometric parameters and investigate the misalignment independent of other factors. Without using additional sensors, the change in the geometric misalignment along the magnification was determined in [18] by capturing a known calibration phantom at different SRDs.

### 1.3. Online Calibration

If the calibration is part of the reconstruction and the system parameters are determined directly using the measurement, the term online calibration is used. A calibration using an object without prior knowledge on its precise geometry was used by Panetta et al. [19] to determine geometric system misalignment. The authors calibrated the system using the projections of a mouse femur and subsequently reconstructed the data using the determined parameters. While the principal point in the *X*-direction and two detector rotations could be estimated, it was not possible to determine the principal point in the *Y*-direction, the detector rotation around the *X*-axis, and errors in the SDD. An adapted cone beam reconstruction based on the FBP reconstruction with the correction system misalignment using six parameters was developed and tested on simulated and real data in [20]. In [21], an iterative Feldkamp–Davis–Kress (FDK)-based reconstruction was described, where the system geometry is considered by describing the trajectory by eight points. The authors achieved similar or better results in comparison with an offline calibration method. Recently, Hofmann et al. [22] implemented and published the source code to a FBP, where the center of rotation and the rotation axis tilt are determined.

### 1.4. Detector Deformation

In addition to geometric misalignment, distortions of the detector can occur in CT systems similar to optical cameras. A mean distortion of a flat panel detector of 0.05 px was found in [23]. Lüthi et al. [14] determined the distortion for different SDDs and separated them into in-plane and off-plane distortions. The total distortion was up to 1.8 px for a SDD of 0.5 m, and the detector tomography showed a difference between high and low spots on the detector of 0.99 mm.

### 1.5. System Instabilities

The majority of publications on the calibration assume a stable geometry with a constant set of parameters. Non-perfect circular rotations were considered in [24,25] for a single photon emission computed tomography system (SPECT). In [26], the authors used Simultaneous Localization And Mapping (SLAM) with SURF feature detection in the X-ray projections for refining the trajectory. A number of parameters were used by Neukamm and Schulze [27] to describe instabilities within the CT geometry, including rotational instabilities during the calibration. Unstable geometries during and between CT measurements can also be caused by temperature changes that could result in the drift of parameters. The drift of the focal spot position of a mobile cone beam tomography system was investigated in [28].

### 1.6. Application of Calibration

To benefit from the determined system parameters describing the misalignment, it has to be considered in the reconstruction. Depending on the software or algorithm used for reconstruction, the parameters can be directly included in the process. In the Reconstruction Toolkit (RTK) [29], a set of eight parameters (+gantry angle) can be used to describe the geometry, either for each projection individually or for all together. The ASTRA Toolbox provides the option to define the geometry for each projection using the vector-based geometry description [30]. The determined geometry parameters can be converted to this description as was shown in [31,32]. TIGRE is another open-source reconstruction toolbox [33] that is able to run CT reconstruction on multiple GPUs and allows to define the system geometry for each projection similar to the ASTRA Toolbox. If the software does not consider geometry misalignment and assumes the perfect CT geometry, new projection images can be calculated based on the determined parameters using image interpolation. Using this, the correction of the detector distortion is possible [14].

### 1.7. Contribution of Study

Currently, the calibration of µCT systems is often controlled by commercial manufacturers of hardware and software or conducted on a research level. In contrast to the multiple open toolboxes for reconstruction, users of µCT systems have, to our knowledge, no options to investigate or calibrate the geometric misalignment of their systems. The main contributions of this publication are, therefore, as follows:

- Implementation of a calibration to determine geometry parameters of a µCT system for the understanding of geometric misalignment;
- Investigation into remaining errors after calibration, particularly the systematic effects caused by the incomplete representation of a µCT device by a geometry model;
- The source code of this work is released to enable further research.

In the following, we first describe the used µCT device, the calibration phantom, and the data capture. Then, we present two mathematical models for tomography systems and illustrate the calibration process. To avoid the necessity of a precise calibration object, the method is designed as a self-calibration approach. In Section 5, we show the results of the validation of the models on the basis of tomography data of a cylindrical helix calibration object acquired by a Procon µCT device.

## 2. Hardware

### 2.1. µCT System

**Figure 2:**
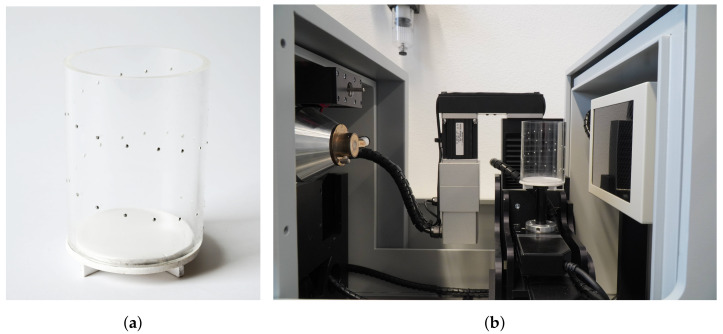
Custom-made calibration phantom with a height of 80 mm and a diameter of 60 mm (**a**) and ProCon CT-XPRESS system with the calibration phantom inside (**b**).

The µCT system employed in this study is the CT-XPRESS manufactured by ProCon X-ray GmbH in Sarstedt, Germany (Figure 2b). The X-ray source has a maximum voltage of 100 kV and a current of up to 200 μA. The flat panel detector has 2940 px by 2304 px with a pixel size of 49.5 μm. The SDD is 353 mm and unchangeable. The rotation axis can be adjusted in the Z-direction, allowing different magnifications, as well as in the *X*- and *Y*-directions for the optimal positioning of the sample.

Note that the µCT system is nominally capable of scanning down to 5 μm and to capture fine details such as cell walls in leaves as shown in [34]. The voxel size achieved in this data set was down to 2 μm, much smaller than the capabilities intended by the manufacturer.

### 2.2. Calibration Phantom

For the test of the implemented methods, a calibration phantom was made using an acrylic glass cylinder and steel ball bearings similar to the process described in [12]. In a helix pattern, 32 small holes were drilled in to the cylinder, in which the ball bearings fixed with glue (Figure 2a). Thirty ball bearings with a diameter of 1.5 mm were used. For differentiation, one larger and one smaller bearing (1 mm and 2 mm) were positioned in the center of the phantom. The exact coordinates of the ball bearings were adjusted during the calibration, thus avoiding the necessity to determine reference values at the micrometer precision level.

### 2.3. Data Capture

**Figure 3:**
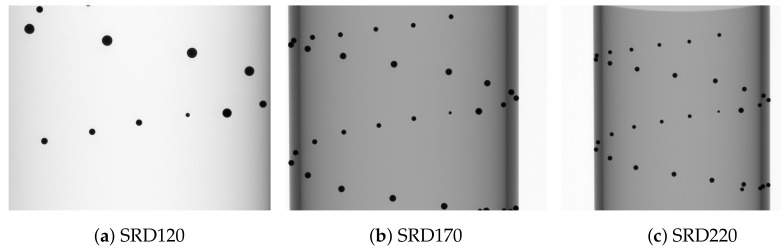
First projection of each data set at the different magnifications.

Multiple data sets of the calibration phantom were captured. For subsequent testing and evaluation, three data sets at different magnifications were used (see Figure 3). At a SRD of 220 mm, the complete calibration phantom was captured, but on the left and right edges of the detector, no measurements accrued (Figure 3c). Measurements on all parts of the detector were possible at a SRD of 170 mm. At a SRD of 120 mm, only the center part of the phantom was depicted on the detector frame. In the following, the three data sets are called SRD120, SRD170 and SRD220.

The same settings were selected for all data sets, and a total of 2161 projections were captured with 100 kV and 49 μA over 360°. The exposure time was 0.45 s and the projections were averaged from three captured images. Binning was disabled, and no filters were used.

## 3. System Geometry

The purpose of the system geometry definition is to describe how a 3D point on the rotation axis is projected onto the detector plane. In contrast to the ideal geometry with just two parameters, a real µCT with misalignment requires more parameters. In this section, two possible ways of defining the geometry of a µCT system are presented. For this, we assume the radiation source to be an infinitely small point and the system geometry to be stable over time. The origin of the pixel coordinate system is at the center of the top left pixel. In this system, the *u*-axis points rightward, while *v*-axis points downward from the origin. The pixel coordinates (u,v) are converted to the detector coordinate system (x,y) with

(1) $$\begin{array}{c} x=\Delta s\left(u-\frac{m}{2}+0.5\right)\;\mathrm{and}\;y=\Delta s\left(\frac{n}{2}-v-0.5\right), \end{array}$$

where *m* is the number of pixels in a row, *n* is the number of pixels in a column, and Δs is the pixel size in mm.

The process of projecting 3D coordinates ${\left[X\;Y\;Z\;1\right]}^{T}$ onto a flat panel detector ${\left[x\;y\;1\right]}^{T}$, often referred to as forward projection, can be accurately modeled using homogeneous coordinates and a 3 × 4 projection matrix *P*. The projection can be represented as

(2) $$s\left[\begin{array}{c} x\\ y\\ 1 \end{array}\right]=P\left[\begin{array}{c} X\\ Y\\ Z\\ 1 \end{array}\right],$$

with *s* being an arbitrary scale and the projection matrix for the ideal geometry

(3) $$P=\left[\begin{array}{ccc} -SDD & 0 & 0\\ 0 & -SDD & 0\\ 0 & 0 & 1 \end{array}\right]\left[\begin{array}{cccc} \cos(\alpha ) & 0 & \sin(\alpha ) & 0\\ 0 & 1 & 0 & 0\\ -\sin(\alpha ) & 0 & \cos(\alpha ) & -SRD \end{array}\right],$$

with α being the gantry angle, which is the angle of the rotation axis for a specific projection. The two distances define the length from the radiation source to the detector (SDD) and the rotation axis (SRD). Based on this ideal geometry definition, the misalignment can be modeled in many different ways. Two possible definitions are described in the following paragraphs. The main difference between them is what parts of the system are fixed and what parameter can be adjusted. In addition to these two described geometries definitions, the model used in RTK was implemented as well.

### 3.1. Definition Using the Detector Plane (Model: “Detector”)

**Figure 4:**
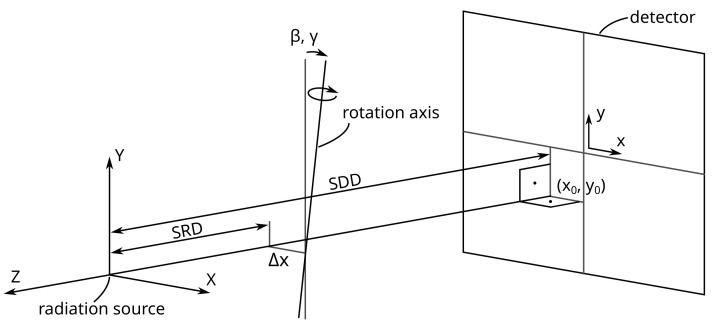
Geometry of the Detector model with the misalignment represented by the principal point, the two rotations, and one translation of the rotation axis.

For the Detector model, the detector and the radiation source are used to define the coordinate system (Figure 4). The optical axis (*Z*-axis) is the shortest distance to the detector plane (SDD) and therefore orthogonal to it. Misalignment, represented by the principal point ($x_{0},y_{0}$), models the deviation between the intersection of the optical axis and the detector center. The remaining misalignment is described by the rotation and translation of the rotation axis. The two rotations are around the *Z*- and *X*-axes (β, γ) and the translation is along the *X*-axis (Δx). These adjustments ensure that the rotation axis intersects correctly with the optical axis. The complete geometry of the CT system under the “Detector” model is thus characterized by seven geometry parameters, SDD, SRD, $x_{0}$, $y_{0}$, β, γ, Δx, and additionally the gantry angle (α).

**Figure 5:**
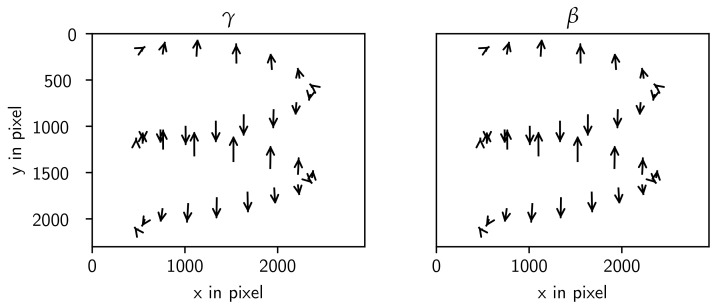
Visualization of the effect of rotation angles for the Detector model. Out-of-plane angle (γ) and in-plane angle (β) rotation of up to 0.25 rad.

The projection matrix for the Detector model is given by

(4) $$P_{Detector}=\left[\begin{array}{ccc} -SDD & 0 & x_{0}\\ 0 & -SDD & y_{0}\\ 0 & 0 & 1 \end{array}\right]\left[\begin{array}{cccc} R_{11} & R_{12} & R_{13} & \Delta x\\ R_{21} & R_{22} & R_{23} & 0\\ R_{31} & R_{32} & R_{33} & -SRD \end{array}\right],$$

where the rotation matrix is

(5) $$R=\left[\begin{array}{ccc} \cos(\beta ) & -\sin(\beta ) & 0\\ \sin(\beta ) & \cos(\beta ) & 0\\ 0 & 0 & 1 \end{array}\right]\left[\begin{array}{ccc} 1 & 0 & 0\\ 0 & \cos(\gamma ) & -\sin(\gamma )\\ 0 & \sin(\gamma ) & \cos(\gamma ) \end{array}\right]\left[\begin{array}{ccc} \cos(\alpha ) & 0 & \sin(\alpha )\\ 0 & 1 & 0\\ -\sin(\alpha ) & 0 & \cos(\alpha ) \end{array}\right],$$

which is composed of individual rotation matrices around the axes representing the gantry angle (α), the in-plane angle (β), and the out-of-plane angle (γ). The result of adjusting the in- and out-of-plane angles on the projection is visualized in Figure 5. A significant disadvantage of this model arises from the high correlation between the parameters $x_{0}$ and Δx, particularly when the rotation axis is near the optical axis. Visualizing the change in the in-plane and out-of-plane angles on the detector shows that the rotation axis is rotated and not the detector (Figure 5). Note that this definition is similar to the geometry model of the RTK library. The source offset in the RTK model is not part of this description, and the translation of the rotation axis Δx is missing in the RTK model.

### 3.2. Definition Using the Rotation Axis (Model: “Tilt”)

**Figure 6:**
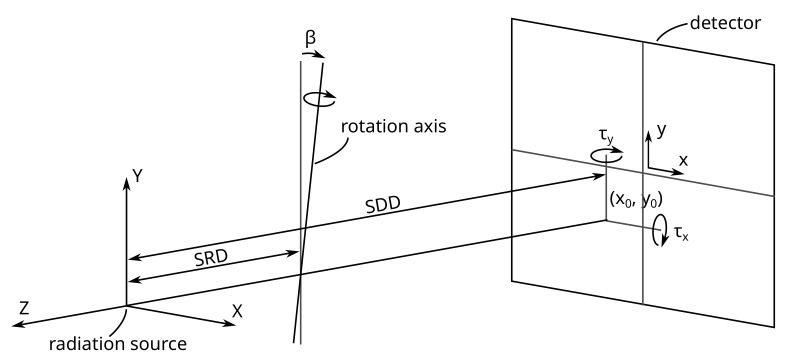
Geometry of the Tilt model with the misalignment represented by the principal point, the two rotations of the detector and an inclination of the rotation axis.

The system geometry can also be defined using the radiation source and the rotation axis, with the shortest distance between these being the SRD, which aligns with the optical axis (*Z*-axis). The rotation axis is parallel to the *Y*-axis. Similar to the Detector model, the principal point ($x_{0},y_{0}$) is used to describe the difference between the center of the detector and its intersection with the optical axis. The other misalignment is modeled as a rotation of the detector. In this case, the intersection between the optical axis and the detector is not orthogonal. The use of a rotation matrix as in Equation (5) would use the *X*-ray source as the center of rotation, which would result in a high correlation to the principal point. To avoid this, the detector is rotated around the principal point (Figure 6).

Generally, tilted sensors are not considered in the standard pinhole model. Tilt–shift lenses, where the optical axis is tilted relative to the sensor, are an exception. The calibration of these lenses has been explored in multiple publications [35,36,37]. One common approach for modeling the tilted sensors can also be found in the open-source computer vision library OpenCV (4.9.0).

The projection matrix for the Tilt model is given by

(6) $$P_{Tilt}=\left[\begin{array}{ccc} -SDD & 0 & x_{0}\\ 0 & -SDD & y_{0}\\ 0 & 0 & 1 \end{array}\right]R_{Tilt}R_{Z}\left[\begin{array}{cccc} \cos(\alpha ) & 0 & -\sin(\alpha ) & 0\\ 0 & 1 & 0 & 0\\ \sin(\alpha ) & 0 & \cos(\alpha ) & -SRD \end{array}\right],$$

where the in-plane rotation around the *Z*-axis is

(7) $$R_{Z}=\left[\begin{array}{ccc} \cos(\beta ) & -\sin(\beta ) & 0\\ \sin(\beta ) & \cos(\beta ) & 0\\ 0 & 0 & 1 \end{array}\right].$$

The detector tilt is based on the calibration of tilt–shift cameras in photogrammetry and computer vision and is implemented as in the OpenCV library with

(8) $$R_{Tilt}=\left[\begin{array}{ccc} R{\left(\tau_{x},\tau_{y}\right)}_{33} & 0 & -R{\left(\tau_{x},\tau_{y}\right)}_{13}\\ 0 & R{\left(\tau_{x},\tau_{y}\right)}_{33} & -R{\left(\tau_{x},\tau_{y}\right)}_{23}\\ 0 & 0 & 1 \end{array}\right]\;\cdot \;R\left(\tau_{x},\tau_{y}\right),$$

where $R(\tau_{x},\tau_{y})$ is defined as

(9) $$R\left(\tau_{x},\tau_{y}\right)=\left[\begin{array}{ccc} \cos(\tau_{y}) & 0 & -\sin(\tau_{y})\\ 0 & 1 & 0\\ \sin(\tau_{y}) & 0 & \cos(\tau_{y}) \end{array}\right]\left[\begin{array}{ccc} 1 & 0 & 0\\ 0 & \cos(\tau_{x}) & -\sin(\tau_{x})\\ 0 & \sin(\tau_{x}) & \cos(\tau_{x}) \end{array}\right].$$

**Figure 7:**
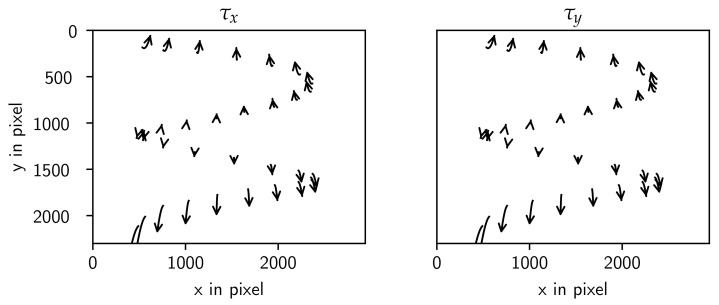
Visualization of effects of $\tau_{x}$ and $\tau_{y}$ from 0 up to 0.7 rad for the Tilt model.

The visualization of the two tilt angles $\tau_{x}$ and $\tau_{y}$ shows the 2D points in the detector frame (Figure 7).

## 4. Implementation

### 4.1. Preprocessing of Projection Images

The captured projections from the CT system were converted from 16-bit RAW to 8-bit image files using *ImageJ* (1.54), and the gray values of each image were normalized to the 8-bit range. For the detection of the ball bearings, a previously developed method for photogrammetric marker detection was used. The process primarily employs Canny edge detection, followed by a search for connected edges, and subsequent ellipse fitting using the connected edge points. Multiple checks and an iterative robust ellipse fitting method [38] remove overlapping ball bearings and outlier edge pixels.

To establish correspondence between the 2D ellipses and the ball bearings, the ellipses were tracked across multiple projections. In the first image, each ellipse was assigned to an ID. In subsequent images, each ellipse was assigned to the ID of the closest matching ellipse from the previous image. If no ellipse was found within a specified distance, a new ID was used. Overlapping ball bearings can cause the tracking to be lost across the data set, resulting in multiple IDs being assigned to each sphere. Detecting and measuring the spheres in one data set with 2161 projections and tracking them could be completed in under a minute utilizing multi-threading (AMD Ryzen 7 3800X). The image coordinates, along with the assigned IDs, were saved in an XML file for further analysis.

### 4.2. System Parameter Adjustment

The adjustment of the unknown system geometry parameters was implemented using the C++ library *Ceres Solver* (2.1.0), which can be used for solving large non-linear least squares problems [39]. The geometry models previously described in Section 3, which project 3D points onto the detector frame, were implemented as cost functions using C++ template functions. The Huber loss function was used to reduce the cost of outliers on the calibration parameters.

Initial values for the unknown parameters are essential for the adjustment process. In all implemented geometry models, most values start at 0, with the exception of SDD and SRD, which are preset. The initial value for Δα is set to $\Delta \alpha =\frac{2\pi }{n_{r}}$, where $n_{r}$ is the number of projections. The following process is used to initialize the 3D coordinates:

1. For each point, two projections are automatically selected, in which the point is visible and for which the angle difference is in a range around 90°. This avoids angles that are not suitable for calculating the 3D coordinates.
2. The projection matrix (*P*) for both projections is calculated using the initial geometry parameters.
3. Using the projection matrices and the two observations in detector coordinates, the 3D coordinates are calculated. For this, the OpenCV (4.8.0) function *triangulatePoints()* is used, which is internally based on singular value decomposition.

The initial 3D coordinates for the ball bearings are adjusted during calibration and therefore do not provide a scale for calibration. To provide a scale to the adjustment, the SRD is kept fixed during calibration. The developed methods permit the use of various types of calibration phantoms, provided that the tracking of the ball bearings is feasible across a range of projections. All other geometry parameters and the gantry angle step are adjusted. Within the calibration process, the adjustment of the unknown parameters is performed iteratively. Between the adjustments, the following processing is used to refine the observations:

- Due to overlapping points and other issues during the detection of the ball bearings, the tracking is lost. This will result in one 3D point having two or more 3D coordinates. After the first calibration, close 3D points are merged using a threshold and the corresponding observations are reassigned.
- Using the projection of the 3D coordinates into the detector frame, observations without an ID are assigned to one.
- Outlier observations are removed by evaluating the residuals using the determined geometry parameters.
- The spheres are imaged as ellipses on the detector plane. An additional correction is performed because the center of the ellipse does not align with the projected center of the sphere. This eccentricity correction is implemented based on the method by [40].

During calibration, an extensive report is generated, allowing the evaluation of the final geometry parameters. The residuals are the difference between the observed and projected coordinates for both axes (*r*). $S_{0}$ is the standard deviation of the residuals and is calculated by

(10) $$S_{0}=\sqrt{\frac{\sum r^{2}}{n-u}},$$

using the number of observations *n* and the number of unknowns *u*. By estimating the covariance matrix *Q* of the adjustment, the standard deviation for the parameter *i* can be estimated with

(11) $$S_{i}=S_{0}\sqrt{Q_{ii}}.$$

The correlation between the two parameters *i* and *j* can also be estimated with

(12) pij=QijQiiQjjQjj.

The different directions of the residuals ($r_{x},r_{y}$) can be evaluated separately with

(13) $$RMSE_{x}=\sqrt{\frac{\sum r_{x}^{2}}{n_{x}}}\;\mathrm{and}\;RMSE_{y}=\sqrt{\frac{\sum r_{y}^{2}}{n_{y}}}.$$

For a combined evaluation, the RMSE of the Euclidean norm is calculated as

(14) $$RMSE_{d}=\sqrt{\frac{\sum \left(r_{x}^{2}+r_{y}^{2}\right)}{n}}.$$

The RMSE can be calculated for all observations or a subset, e.g., only using observations of one projection or one ball bearing. The implementation offers flexibility, allowing for the addition of new geometry models or adjustment of parameters to control various aspects of the process. The use of the determined geometry parameters in reconstruction is not explored in this publication.

## 5. Results

In the following section, the results of the calibrations are presented, starting with the adjusted parameters and the RMSE of the residuals. Additionally, the correlation between the parameters and remaining systematic effects is investigated.

### 5.1. Parameters

Calibrations were performed for all three data sets using both previously defined geometry models. All parameters were adjusted except for the SRD, which was fixed to provide a scale. In the Detector model, the misalignment is described as rotation of the rotation axis around the *Z*-axis (β) and the *X*-axis (γ), as well as the translation of the rotation axis (Δx). The parameters and their standard deviations, and additional results of this model, are shown in Table 1. The $RMSE_{d}$ value is 0.27 px for SRD120 and decreases to 0.26 px for SRD170 and 0.18 px for SRD220. Note that for SRD120 $y_{0}$, γ and SDD differ significantly from the other data sets. Additionally, the *Y*-coordinates of the adjusted object points for this data set are around 16 mm higher than from the points of the other data sets. Assuming a perfect system geometry, where the geometry parameters are fixed at their initial values and only the object points are adjusted, the $RMSE_{d}$ is 10.61 px for SRD120, 63.35 px for SRD170, and 78.83 px for SRD220.

In the Tilt model, the misalignment is modeled by the inclination of the rotation axis around the *Z*-axis (β), similar to the Detector model. There are two further rotations that were applied to the detector ($\tau_{x},\tau_{y}$). The RMSE is similar between the two models (Table 2). Due to the outlier removal, the number of image points used for calibration varies between the two models. The influence of integrated eccentricity correction is minimal and depends on the magnification. For the Tilt model, the mean correction of an observation was 0.039 px for SRD120 and 0.0097 px for SRD220.

### 5.2. Correlation

**Figure 8:**
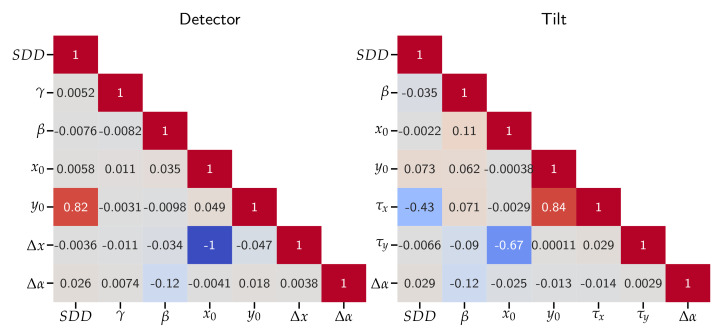
Correlation coefficients between the adjusted parameters for SRD220 of both geometry models.

The correlation matrices for the different implemented models illustrate the dependencies among the geometry parameters introduced as unknowns (Figure 8). For the Detector model, a high correlation close to −1 (−0.99995) between Δx and $x_{0}$ occurs. If a high correlation is observed between two parameters, one of them can be disabled. For SRD220, disabling Δx will increase $RMSE_{d}$ to 0.24 px. If $x_{0}$ is not adjusted, $RMSE_{d}$ will increase to 0.52 px. In the Tilt model, the highest correlation, though lower than that in the Detector model, occurs between $\tau_{x}$ and $y_{0}$, with a coefficient of 0.84. A negative correlation of −0.67 is observed between $x_{0}$ and $\tau_{y}$. For both models, the parameters of the principal point exhibit interactions with other parameters. In summary, the Tilt model shows lower overall correlations compared to the Detector model. The correlation for the other data sets can be found in Appendix A.1. Note the high correlation of −0.99 in SRD120 between SDD and $y_{0}$, which could indicate why the resulting parameters differed from the other data sets.

### 5.3. Residuals

**Figure 9:**
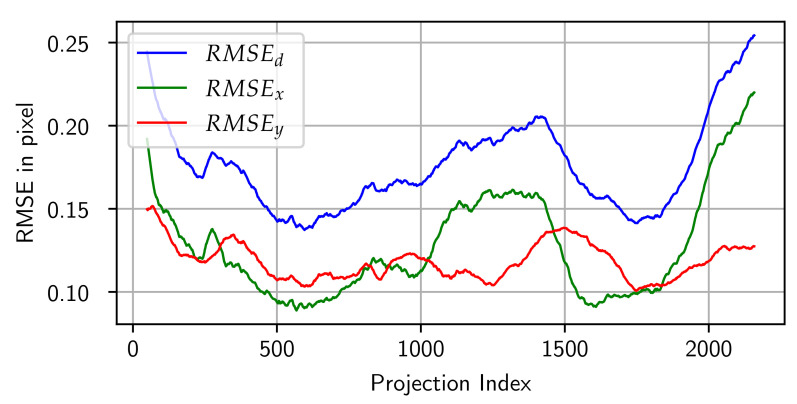
Moving average (window = 50) of the RMSE of the residuals over all projections for the data set SRD220.

The residuals for each observation between the implemented geometry models only differ slightly. The maximum absolute difference between the residuals of the two models for SRD220 is 3.1 × 10^−4^ px. Therefore, only the residuals of the Tilt model are used in the following investigations. To examine the stability of the device geometry, the RMSE of the residuals can be calculated for each projection. Figure 9 shows the moving average of the RMSE over all projections in *X*- and *Y*-direction as well as for the Euclidean norm. Variations can be observed in both directions but are larger for $RMSE_{x}$ than for $RMSE_{y}$, where large errors occur at the start and end of the data capture.

**Figure 10:**
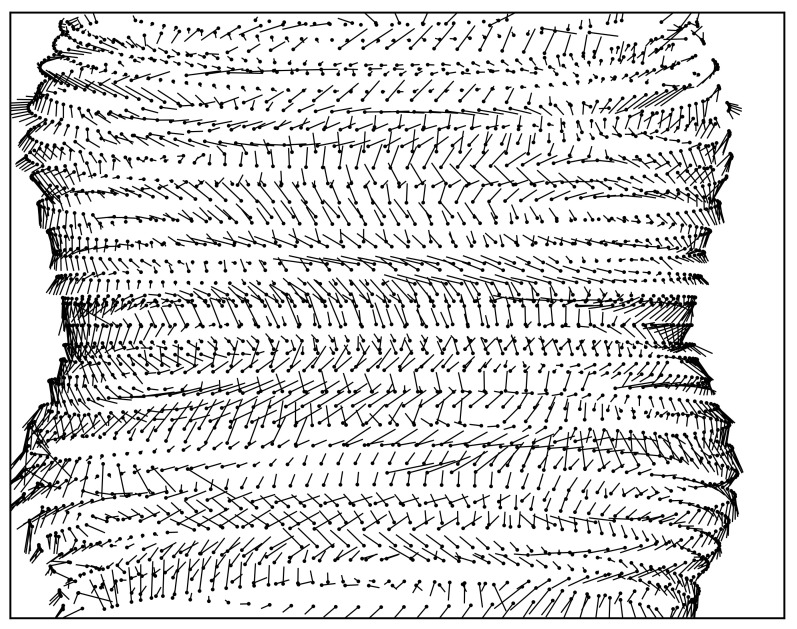
Plot of the 250-magnified residuals for SRD170 that remain after calibration. For visualization, the residuals are averaged over 20 projections, i.e., for each point, an average residual is plotted at an average coordinate. Note that some lines of residuals are cut off at the image edge.

**Figure 11:**
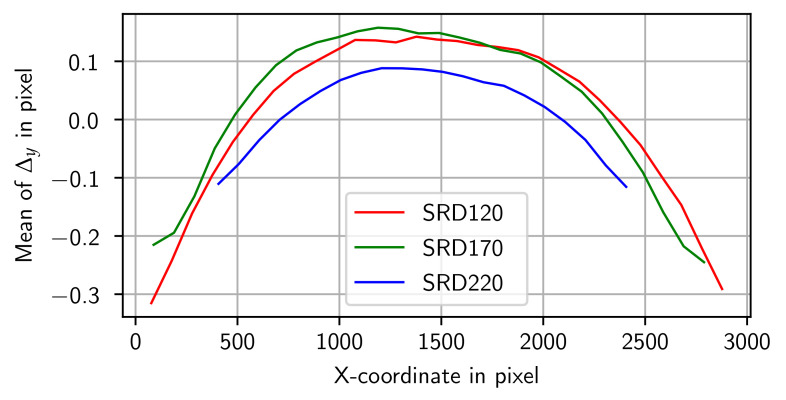
Mean of the *Y*-residuals (Δy) in pixels with a window size of 100 in relation to the *X*-coordinate for all three data sets.

Plotting the residuals after the calibration may support determining the remaining systematic errors in the model. Figure 10 shows the residuals for SRD170. One global systematic effect that can be observed is in the *Y*-direction. The magnitude and direction of the error depend on the *X*-position on the detector. While in the center of the detector, the error is pointing to the top. On the left and right edges, the orientation is towards the bottom edge. This is also observable by grouping the residuals into bins along the *X*-axis with a width of 100 px and calculating the average of the *Y*-residual for each bin. The effect is visible in all three data sets but with different magnitudes and ranges (Figure 11).

### 5.4. System Instability

**Figure 12:**
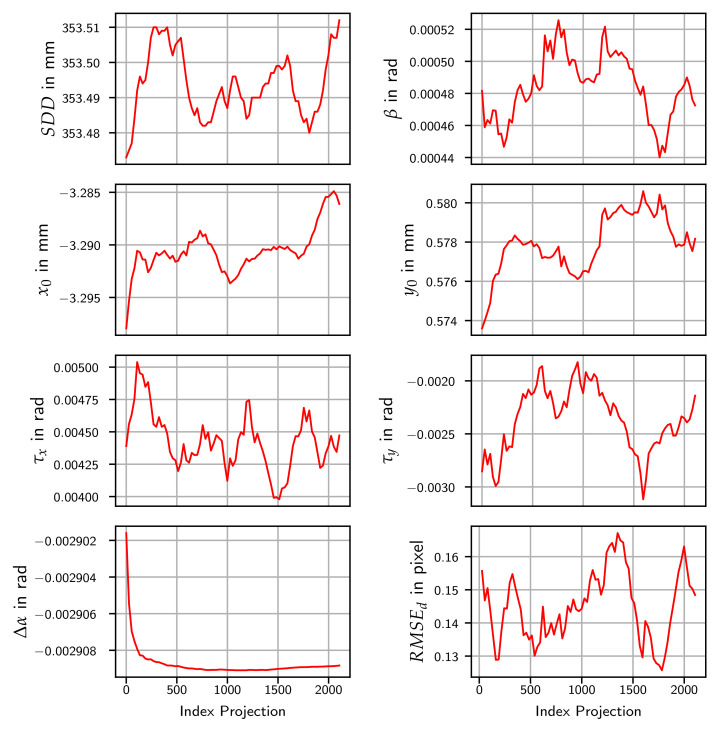
Resulting parameters and RMSE after calibrations using blocks of 27 consecutive projections for the Tilt model on SRD220.

Due to the systematic effects, an experiment to investigate the possibility of an unstable system geometry and changing parameters during data capture was carried out. For this, the SRD220 data set was split in 80 blocks, each containing 27 consecutive projections. For the experiment, the observations of each block were used to adjust the model parameters. The 3D coordinates of the object points were adjusted beforehand using all projections and kept fixed in this experiment. The result of the Tilt model is shown in Figure 12. All calibration parameters show systematic changes over the projections. A drift of the values for the principal point ($x_{0},\;y_{0}$) of up to 0.01 mm (0.2 px) can be observed. The gantry angle step (Δα) shows a clear change for the first calibrations. The angle between two successive projections is increasing at the beginning and then stabilizing. For the Detector model, the results can be found in Appendix A.2.

## 6. Discussion

Open tools to investigate the system geometry of µCTs, which are independent of the manufacturer, are, to the best knowledge of the authors, not available at the moment. In contrast to the reconstruction of computed tomography images, where multiple tools are available, black box solutions of the manufacturers are the standard for calibration. Thus, the source code for this study is published as a first step.

The developed and implemented method can detect steel ball bearings on a calibration phantom in the projections and use these observations to adjust system geometry parameters to fit a model. The method was tested on multiple data sets of a µCT system and is capable of determining the geometry parameters. Using the calibration, the $RMSE_{d}$ decreases for SRD220 from 78.83 px to 0.18 px. The remaining errors after calibration are the same for all implemented geometry models. This error after calibration indicates how well the model represents the data. Remaining errors in the detector are at the sub-pixel level, which show multiple systematic effects and depend on the magnification of the system. For large magnifications with a SRD of 120 mm, the $RMSE_{d}$ is 0.27 px, significantly higher than at a SRD of 220 mm with 0.18 px. A possible reason for this could be that changing system geometries, such as the drifting of the radiation source, have a greater impact at higher magnifications [4].

Although all implemented models result in the same residual errors, the correlation matrix shows that the Tilt model has more independent parameters than the Detector model. High correlations between system parameters may result in a calibration that fits well to the calibration data but does not model the real system geometry. In photogrammetry, reducing the correlation between the parameters is achieved by capturing the calibration objects at specific orientations. For µCT systems, similar results might be achieved by capturing the calibration phantom at different positions of the rotation axis or rotating it around the *X*- or *Z*-axis.

For data set SRD120 and the Detector model, significant deviations of some parameters ($y_{0}$, γ, and SDD) and high correlation between SDD and $y_{0}$ are observed. The reason for this cannot be determined, but we assume that due to the high correlation, a different minimum is found during the adjustment. If this is the case, the parameters obtained may not be applicable to other data sets.

The remaining systematic residuals on the detector in the *Y*-direction show that geometry models alone are not able to fully represent the misalignment of the used µCT system. Possible causes for this could be distortions of the detector. However, these would result in constant errors depending on the location on the detector over multiple data sets. The variation of the error over the projections within one data set indicates that other or additional effects are present in the data. If blocks of projections are used for calibration, the changing parameters over one data set also imply that either the geometry changes or is not fully modeled. Due to the overlapping effects, determining the causes of the occurring systematic errors remains a challenge. One possible cause could be the geometric instability over the recording time, for example, such as a drifting radiation source or other temperature-related changes in the system. For this work, we also assume temporal stability of the calibration phantom during the CT scan. But due to a temperature increase in the system during the capture process the phantom will heat up and increase in size.

The investigation shows that further research is necessary, including the test of the method on other CT systems. Open questions include whether determined parameters can be applied to other samples, and the stability of the system geometry and thus also of the calibration. In the case of geometrically unstable systems, the accuracy of the used geometry parameters must be checked in regular intervals. If a reduced accuracy is observed, a re-calibration may be necessary. Due to the flexibility of µCT systems and the resulting complexity of their calibration, methods integrated with the reconstruction to determine the geometry are desirable. This would reduce the need for separate calibrations.

## Figures and Tables

**Table 1:** Results of the calibration using the Detector model across all data sets, showing model parameters with their standard deviations.

	SRD120	SRD170	SRD220
$RMSE_{d}$ in px	0.27	0.26	0.18
Mean Eccentricity in px	0.055	0.02	0.0098
$N_{Image\;points}$	23,884	56,614	64,589
$N_{Object\;points}$	24	32	32
SDD in mm	349.56 ± 0.0025	353.44 ± 0.0027	353.49 ± 0.0025
γ in rad	−0.15 ± 9.8 × 10^−7^	0.004 ± 9.3 × 10^−7^	0.0052 ± 7.2 × 10^−7^
β in rad	0.00088 ± 1.3 × 10^−6^	0.00063 ± 7.7 × 10^−7^	0.00048 ± 5.8 × 10^−7^
$x_{0}$ in mm	−2.63 ± 0.0036	−2.54 ± 0.0026	−2.48 ± 0.0026
$y_{0}$ in mm	−51.81 ± 0.00035	2.03 ± 5.7 × 10^−5^	2.43 ± 4 × 10^−5^
Δx in rad	−0.32 ± 0.0012	−0.41 ± 0.0012	−0.5 ± 0.0016
Δα in rad	−0.0029 ± 1.7 × 10^−9^	−0.0029 ± 1.5 × 10^−9^	−0.0029 ± 1.2 × 10^−9^

**Table 2:** Results of the calibration using the Tilt model across all data sets, showing model parameters with their standard deviations.

	SRD120	SRD170	SRD220
$RMSE_{d}$ in px	0.27	0.26	0.18
Mean Eccentricity in px	0.039	0.02	0.0097
$N_{Image\;points}$	23,881	56,613	64,588
$N_{Object\;points}$	24	32	32
SDD in mm	353.46 ± 0.0026	353.44 ± 0.0027	353.49 ± 0.0025
β in rad	0.00049 ± 1.8 × 10^−6^	0.00064 ± 7.7 × 10^−7^	0.00049 ± 5.9 × 10^−7^
$x_{0}$ in mm	−3.53 ± 8.9 × 10^−5^	−3.39 ± 5.8 × 10^−5^	−3.29 ± 3.4 × 10^−5^
$y_{0}$ in mm	0.55 ± 0.00033	0.61 ± 0.00032	0.58 ± 0.00026
$\tau_{x}$ in rad	−0.15 ± 1.8 × 10^−5^	0.0014 ± 2.9 × 10^−5^	0.0044 ± 2.7 × 10^−5^
$\tau_{y}$ in rad	−0.0026 ± 1 × 10^−5^	−0.0024 ± 7.3 × 10^−6^	−0.0023 ± 7.2 × 10^−6^
Δα in rad	−0.0029 ± 1.7 × 10^−9^	−0.0029 ± 1.5 × 10^−9^	−0.0029 ± 1.2 × 10^−9^

**Figure A1:**
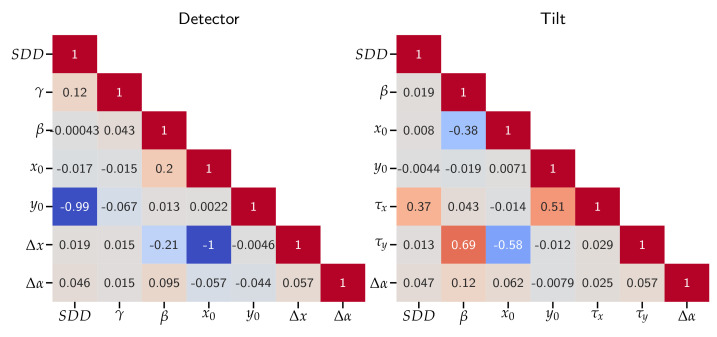
Correlation coefficients for SRD120.

**Figure A2:**
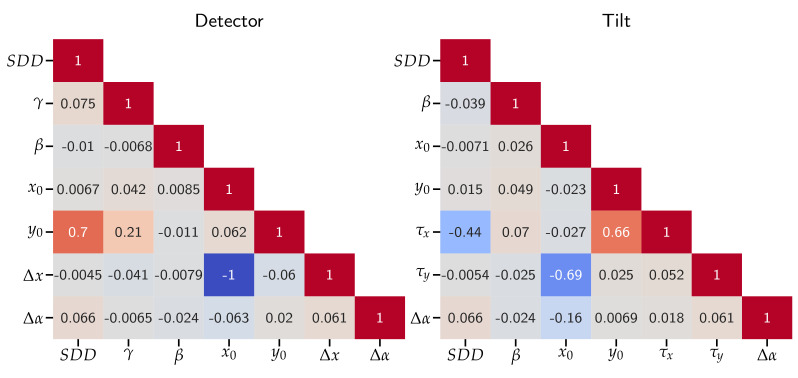
Correlation coefficients for SRD170.

**Figure A3:**
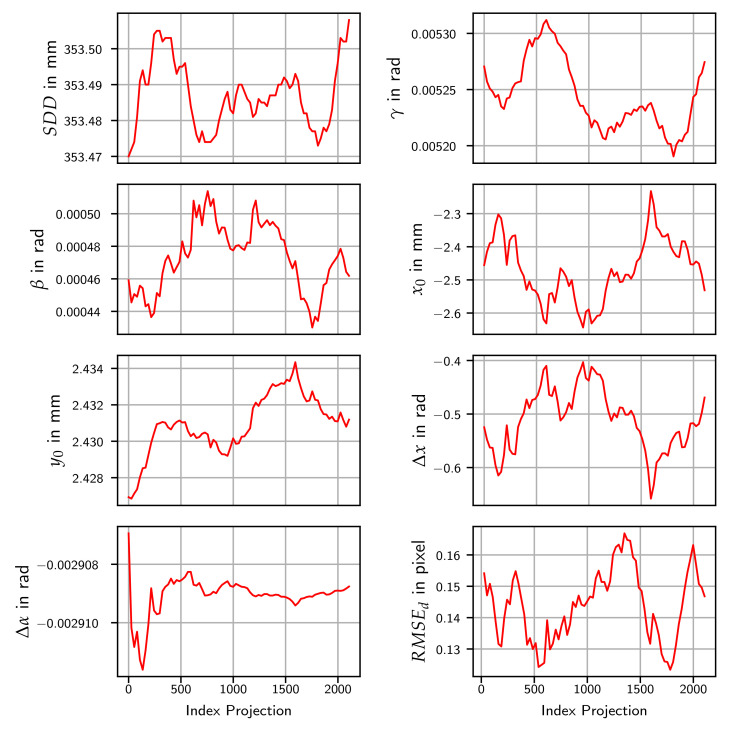
Resulting parameters and RMSE after calibrations using blocks of 27 consecutive projections for the Detector model on SRD220.

## Data Availability

The source code of the calibration is available at https://github.com/matthiashar/CtCalib. The µCT data sets are available at request.

## Abbreviations

µCT	Micro-Computed Tomography
FBP	Filtered Back Projection
FDK	Feldkamp–Davis–Kress
RTK	Reconstruction Toolkit
SDD	Source Detector Distance
SRD	Source Rotation Axis Distance
